# Socio-demographic differences in access to psychological treatment services: evidence from a national cohort study

**DOI:** 10.1017/S0033291723001010

**Published:** 2023-11

**Authors:** Emma Sharland, Klaudia Rzepnicka, Dorothee Schneider, Katie Finning, Piotr Pawelek, Rob Saunders, Vahé Nafilyan

**Affiliations:** 1 Office for National Statistics, Newport, UK; 2 Research Department of Clinical, Educational & Health Psychology, Centre for Outcomes Research and Effectiveness (CORE), University College London, London, UK; 3 Department for Medical Statistics, London School of Hygiene and Tropical Medicine, London, UK

**Keywords:** talking therapies, Improving Access to Psychological Therapies, common mental disorders, mental health, service access

## Abstract

**Background:**

Since 2008, the Improving Access to Psychological Therapies (IAPT) programme has offered adults in England evidence-based psychological treatments for common mental disorders (CMDs) such as depression and anxiety disorders. However, inequalities in access have not been explored at the national level.

**Methods:**

Using a unique individual patient dataset that linked 2011 Census information of English residents to national IAPT data collected between April 2017 and March 2018, we estimated the rate of access by a wide range of socio-demographic characteristics that are not routinely available. A large household survey was used to estimate the prevalence of probable CMDs by these socio-demographic characteristics. We estimated the probability of access to IAPT amongst people with CMDs by comparing the rates of access from IAPT data and the estimates of prevalence of CMDs from the household survey. Both unadjusted and adjusted (for important patient characteristics) access rates were estimated in logistic regression models.

**Results:**

As a proportion of those with a probable CMD, access to IAPT varied markedly by socio-demographic characteristics. Older adults, males, people born outside of the UK, people with religious beliefs, people from Asian ethnic backgrounds, people reporting a disability and those without any academic or professional qualifications were underrepresented in IAPT services nationally, in adjusted models.

**Conclusions:**

The identification of patients who may be underrepresented in IAPT provides an opportunity for services to target outreach and engagement with these groups. Further understanding of barriers to access should help increase equity in access.

## Introduction

In England, one in six adults are estimated to have a common mental disorder (CMD) (McManus, Bebbington, Jenkins, & Brugha, 2016) such as depression or an anxiety disorder. Improving the mental health of the nation continues to be a priority for the UK Government and the National Health Service (NHS) (NHS England, 2019). Launched in 2008, the Improving Access to Psychological Therapies (IAPT) programme offers adults in England evidence-based talking therapies for CMDs. By 2017, nearly 1 million patients were seen by services per year (Clark, 2018). However, for such services to achieve their aim in improving the mental health of the national, equality in access across patient groups is needed. This is especially important given that the burden of CMDs disproportionally affects those from vulnerable backgrounds and lower socio-economic backgrounds, such as ethnic minority groups, disabled people, migrants, people living in deprived areas or those with low educational attainment (Fryers, Melzer, Jenkins, & Brugha, 2005; NHS England, 2016; Nunes et al., 2016).

Previous research from a range of mental healthcare settings suggests there exists inequalities in access. For example, individuals for minoritised ethnicities have been found to be less likely to receive support from mental health services than those from White groups (Simpson, Krishnan, Kunik, & Ruiz, 2007; Van Voorhees, Walters, Prochaska, & Quinn, 2007) and older adults have been identified as particularly disadvantaged, at least historically (Department of Health, 2001). Other factors such as language barriers (Dixon-Woods et al., 2005), religious involvement (Harris, Edlund, & Larson, 2006) and level of educational qualification (Weich, Nazareth, Morgan, & King, 2007) have also been associated with differential access to mental health treatments. However, there has been limited research into inequalities in access to IAPT services, with existing evidence provided by a handful of services indicating that there are some local level inequalities in access. For example, individuals from minority ethnic groups are less likely to be assessed and treated in IAPT, compared to White British individuals (Ahmad, McManus, Cooper, Hatch, & Das-Munshi, 2022; Harwood et al., 2021). Recent analysis of male users from two services in London suggests ethnicity, religious affiliation and socio-economic status are associated with service use (Smyth et al., 2022). However, this has not been explored at the national level. One limitation with using data from IAPT services for such analyses is that some socio-demographic characteristics, such as age, sex, disability, ethnicity, and religion, are collected at the point of registration, but the proportion of valid responses provided nationally are low for some characteristics [ethnicity; 86.5%, religion; 63.9% (NHS Digital, 2018)]. Additionally, several characteristics which may impact mental health or access to services, are not collected during the IAPT registration (e.g. individual measures of socio-economic status). Estimating rate of access to IAPT by socio-demographic characteristics is difficult because of the lack of suitable population data. Finally, to date there has been no attempt to understand how representative the IAPT population is compared to the general population with CMDs. Identifying groups with lower relative access to IAPT could help identify subgroups currently underrepresented in IAPT and support targeted outreach initiatives.

In this study, we examined the socio-demographic characteristics of individuals treated nationally in the IAPT programme and whether participants were representative of the population experiencing CMDs in England. Using a unique individual patient dataset that linked 2011 Census information of English residents to data from all patients seen by the national IAPT programme, we estimated the rate of access by a wide range of socio-demographic characteristics. Using data from a large household survey, we estimated the prevalence of probable CMDs by socio-demographic characteristics. To identify groups which had lower access to IAPT, we estimated the probability of access amongst people with CMDs by comparing the rates of access to IAPT and the estimates of prevalence of CMDs.

## Methods

### Data and participants

We conducted a retrospective cohort study using a unique dataset based on the 2011 Census linked to nationally collected IAPT data and comparing it to data from the UK Household Longitudinal Study (UKHLS) (University of Essex: Institute for Social and Economic Research, 2022). The census for England and Wales is a survey collected every 10 years by Office for National Statistics which provides in-depth information on all people and households in England and Wales (Office for National Statistics, 2022). All IAPT services, which operate across all regions of England, collect the same standardised minimum dataset (the IAPT MDS) and provide monthly submissions of this data to NHS Digital where it is compiled for both monthly and annual reports (John et al., 2022). For the current analysis the IAPT national data for the financial year 2017/18 was used, including all participants who had at least one treatment session as determined by the ‘APPTYPE’ (Appointment type) variable. For this study, we focused on the 2017/18 financial year, the most recent year for which national data were available to the research team and provided by NHS Digital (data owners) for analysis purposes via the Data Access Request Service.

The linkage of IAPT data to the 2011 Census for England and Wales was conducted using NHS number. To obtain NHS numbers for census participants, the 2011 information was linked to the 2011–2013 NHS Patient Registers. Of the 53 483 502 census records, 50 019 451 were linked deterministically to the patient register. 555 291 additional matches were obtained using probabilistic matching (overall linkage rate of Census to NHS Patient Register: 94.6%). The cohort was also linked to death registration data using NHS number, to remove participants who died during the reporting periods. Study participants are the adult population in England aged 18 (at the start of the reporting period) to 100 years, who were usual residents in England when they were enumerated in the 2011 Census. Participants living in Wales at the time of 2011 Census were excluded from the study.

Out of the 1 009 035 IAPT referrals received within the 2017/18 reporting period, we identified 941 008 unique patients. Out of the 941 008 individuals, 766 829 (81.5%) were successfully linked to the 2011 Census (online Supplementary Fig. S1).

We used data from UKHLS waves 8 to 10, to measure probable CMDs within the population at an equivalent time to the IAPT data. The UKHLS data was accessed via the UK Data Archive with addition of lower super output area information requested through the special licence. Interviews for which sample months were between April 2017 and March 2018 were pooled into a financial year 2017/18 dataset equivalent to the IAPT reporting period. Survey weights were scaled to adjust for reduced likelihood to respond over time following UKLHS guidance (Kaminska & Lynn, 2019). The UKHLS includes a representative UK sample with a total sample size of between 50 994 (wave 1) and 34 318 (wave 10) participants per wave. Study participants from the UKHLS were the adult population in England (per financial year) aged 18 to 100 years who were living in the UK in 2011 who responded to the survey. Of the 19 135 respondents meeting the study criteria 3410 were removed because of missing covariate information and a further 111 because of missing outcome. Inverse probability weighting was used to adjust for selection bias due to this item missingness. Sociodemographic characteristics of UKHLS were used from the interview provided closest to March 2011 to match information from census as closely as possible.

### Variables

Our exposures of interest were socio-demographic characteristics potentially related to CMDs or access to healthcare. The burden of CMDs disproportionally affects those from vulnerable backgrounds and lower socio-economic backgrounds, such as ethnic minority groups, disabled people, migrants, people living in deprived areas, those with lower educational attainment (Das-Munshi, Leavey, Stansfeld, & Prince, 2012; Esch et al., 2014; Fone et al., 2014; Fryers et al., 2005; Mercer & Watt, 2007; Naylor et al., 2012; NHS England, 2016; Nunes et al., 2016). Whilst religion may offer some comfort with those with mental health problems (King, Weich, Nazroo, & Blizard, 2006; McCullough & Larson, 1999), some studies suggest religion can be a barrier affecting access to mental health services and psychological therapies (Loewenthal, Mohamed, Mukhopadhyay, Ganesh, & Thomas, 2012).

All individual level socio-demographic characteristics for the access to IAPT analysis came from the 2011 Census data and include age, country of birth, disability status, English as first language, ethnicity, index of multiple deprivation (IMD), qualifications, region of England, religious affiliation, and sex (See online Supplementary Table S1). To estimate the prevalence in the general population, the socio-demographic characteristics came from the UKHLS interviews that were available closest to the 2011 Census date (±26 months) or the first adult interview for someone aged 16 years.

### Outcome measures

For the analysis of access to IAPT, the main outcome was having received at least one treatment session in IAPT between April 2017 and March 2018.

For the analysis of the prevalence of CMDs using the UKHLS, the main outcome was having a General Health Questionnaire (GHQ)-12 score of 4+ using the standard scoring method to proxy probable CMD.

GHQ-12 is a validated measure that is commonly used to estimate non-psychotic psychiatric morbidity and CMDs (Brown, Harris, Srivastava, & Taylor, 2018; Endsley, Weobong, & Nadkarni, 2017; Stochl, Böhnke, Pickett, & Croudace, 2016). GHQ-12 has been validated against ICD-10 diagnoses (current depression, dysthymia, agoraphobia, panic disorder, generalised anxiety disorder, somatisation disorder, neurasthenia and hypochondriasis) that have large overlap with those covered by IAPT and which has been shown to be as effective as longer screening instruments for mental health for identifying CMDs (Goldberg et al., 1997).

We then compare the two set of rates to estimate the proportion of individuals with CMD who received treatment in IAPT.

### Statistical methods

Descriptive statistics for all variables were calculated in both IAPT and UKHLS cohorts. Data is presented as counts and percentages.

Adjusted rates of the respective outcome measures were derived by calculating marginal probabilities from logistic regression models. For each exposure, we fitted logistic regression models to estimate the probability of receiving treatment (IAPT) and of having a CMD (UKHLS, survey weighted). We fitted unadjusted models and models adjusted for all other exposures using our 18+ population. We restricted the population to individuals aged 25+ when estimating the effects of qualifications and National Statistics Socio-Economic Classification (NS-SEC) to minimise the influence of measurement error between the Census and IAPT time points.

In the UKHLS sample, we used analysis weights for the self-completion questionnaire to adjust for differences in selection probabilities and non-response. We deleted observations with missing data and used inverse probability weighting based on a logistic regression model to account for non-randomness of the missing data.

We used an indirect method to estimate the access to IAPT amongst people with probable CMDs. We estimated the proportion of individuals with CMD who received treatment in IAPT, by dividing the rates of people receiving treatment through IAPT services by the self-reported CMD rates. If for a particular group, the rate of access to IAPT was 3%, and the self-reported CMD rate was 15%, then the rate of access to IAPT among people with probable CMDs was 20% (0.03/0.15). To obtain 95% confidence intervals, we ran a Monte Carlo simulation with one million iterations, drawing each rate from a normal distribution with a mean equal to the estimated rate and a standard deviation equal to the standard error and calculating the ratio. The 2.5th and 97.5th percentiles of the resulting distributions were used to estimate the 95% confidence intervals (Buckland, 1984).

All IAPT, 2011 Census and Mortality data were analysed in R version 3.5.1. All UKHLS data was analysed in STATA version 15.1. The comparison of IAPT to UKHLS was completed in R version 4.1.3.

## Results

### Study populations

The general Census 2011 population for this study was 37 899 905, of which 727 802 (1.9%) received at least one treatment session through IAPT services (around 1 in 50) in 2017/18. The UKHLS cohort comprised 15 614 participants of which 18.1% had a probable CMD.

Our study population (Table 1) were predominantly female (Census: 52.3%; UKHLS: 51.7%), with a mean age 49.8 (±18.6 years) in census and 51.3 years (±18.2 years) in UKHLS, born in the UK (Census: 85.7%; UKHLS: 89.9%), had English as their first language (Census: 92.4%; UKHLS: 94.0%) and White (Census: 87.5%; UKHLS: 91.07%).

**Table 1.** Socio-demographic characteristics of the study population

Variable	Level	Census study population		UKHLS study population	
		Count	%	Count	% (weighted)
Age	18–24	4 040 728	10.7	571	8.8
	25–34	5 834 835	15.4	1295	13.6
	35–44	6 066 477	16	2420	15.3
	45–54	7 021 445	18.5	3202	18.1
	55–64	5 950 640	15.7	3168	17.8
	65–100	8 985 780	23.7	4958	26.4
Country of Birth	Born outside the UK	5 402 703	14.3	1927	10.1
	Born within the UK	32 497 202	85.7	13 687	89.9
Disability Status	Not disabled	31 751 323	83.8	12 446	79.9
	Disabled	6 148 582	16.2	3168	20.1
English as a first Language	English is the first language	35 030 053	92.4	14 380	94.0
	English is not the first language	2 869 852	7.6	1234	6.0
Ethnicity	Asian	2 754 331	7.3	1281	5.1
	Black	1 107 808	2.9	505	2.1
	Mixed	575 867	1.5	252	1.4
	Other ethnic group	294 002	0.8	87	0.4
	White	33 167 897	87.5	13 489	91.1
IMD (quintile)	1 – most deprived	7 003 193	18.5	2712	18.3
	2	7 491 225	19.8	2917	18.9
	3	7 747 955	20.4	3184	20.9
	4	7 810 046	20.6	3339	20.8
	5 – least deprived	7 847 486	20.7	3462	21.1
	1. Management and professional	11 300 409	33.4	5610	38.7
NS-SEC^a^	2. Intermediate	4 771 433	14.1	1920	13.0
	3. Small employers and own account	3 351 001	9.9	1136	8.4
	4. Lower supervisory and technical	2 455 154	7.3	871	7.2
	5. Semi-routine and routine	8 879 907	26.2	3295	25.3
	Never worked or long-term unemployment	1 761 900	5.2	529	4.0
	Missing or not applicable	1 339 373	4	308	3.4
Qualifications^a^	Below a degree level	16 991 085	50.2	6560	50.5
	Degree level or above	10 113 810	29.9	5676	38.2
	No academic or professional qualifications	6 754 282	19.9	1433	11.3
Region	East	4 260 412	11.2	1841	11.7
	East Midlands	3 315 258	8.7	1545	8.8
	London	5 448 081	14.4	1935	15.0
	North East	1 891 092	5	801	5.2
	North West	5 055 813	13.3	2034	12.7
	South East	6 262 362	16.5	2508	15.6
	South West	3 880 860	10.2	1732	10.3
	West Midlands	4 004 770	10.6	1598	10.1
	Yorkshire and the Humber	3 781 257	10	1620	10.7
Religion	Religious affiliation	26 140 718	69	8597	50.0
	No religious affiliation	9 330 385	24.6	7017	50.0
Sex	Female	19 802 724	52.3	8868	51.7
	Male	18 097 181	47.7	6746	48.3

NS-SEC, National Statistics Socio-Economic Classification; IMD, Index of Multiple Deprivation.

a Using a 25 + population.

### Main findings

The proportion of people with a CMD in UKHLS; the proportion of people accessing IAPT services; and those accessing IAPT as a proportion of those with a probable CMD broken down by socio-demographic characteristic can be found in Tables 2 and 3. Findings for qualifications and NS-SEC can be found in Table 4.

**Table 2.** Proportion of individuals with a probable Common Mental Disorder (CMD) defined by a score of 4 and above on the GHQ-12 (A) and proportion of individuals treated in Improving Access to Psychological Therapies (IAPT) (B) broken down by socio-demographic characteristics

	A		B	
Exposure	Model 1^a^% (95% CI)	Model 2^b^% (95% CI)	Model 1^a^% (95% CI)	Model 2^b^% (95% CI)
Age				
18–24	22.9 (18.7–27.2)	24.3 (19.8–28.7)	3.3 (3.2–3.3)	3.3 (3.2–3.3)
25–34	23.2 (20.1–26.3)	24.0 (20.6–27.4)	3.0 (2.9–3.0)	3.0 (2.9–3.0)
35–44	20.4 (18.4–22.4)	21.5 (19.4–23.7)	2.4 (2.3–2.4)	2.4 (2.4–2.4)
45–54	21.5 (19.8–23.2)	20.9 (19.2–22.6)	1.9 (1.9–1.9)	1.8 (1.8–1.8)
55–64	20.0 (18.3–21.7)	18.1 (16.5–19.8)	1.5 (1.4–1.5)	1.3 (1.3–1.3)
65 and over	13.8 (12.7–14.9)	10.9 (9.9–11.9)	0.7 (0.6–0.7)	0.5 (0.5–0.5)
Country of birth				
Born outside the UK	17.0 (14.6–19.4)	17.5 (14.5–20.6)	1.2 (1.2–1.3)	1.3 (1.2–1.3)
Born within the UK	19.6 (18.7–20.6)	18.0 (17.1–18.9)	2.0 (2.0–2.0)	1.7 (1.7–1.7)
Disability				
Disabled	31.6 (29.5–33.8)	33.7 (31.2–36.1)	2.2 (2.1–2.2)	2.7 (2.6–2.7)
Not disabled	16.4 (15.5–17.3)	15.1 (14.3–15.9)	1.9 (1.9–1.9)	1.5 (1.5–1.5)
English as first language				
English first language	19.6 (18.7–20.5)	18.3 (17.4–19.2)	2.0 (2.0–2.0)	1.7 (1.7–1.7)
English not first language	15.4 (12.7–18.1)	13.3 (9.9–16.8)	1.1 (1.0–1.1)	1.1 (1.0–1.1)
Ethnicity				
Asian	18.5 (15.4–21.5)	18.6 (14.6–22.6)	1.3 (1.3–1.4)	1.2 (1.2–1.2)
Black	21.8 (15.1–28.5)	19.3 (13.3–25.3)	1.7 (1.7–1.8)	1.4 (1.4–1.4)
Mixed	30.4 (21.9–38.8)	26.7 (18.6–34.8)	2.9 (2.8–3.1)	1.8 (1.8–1.9)
Other ethnic group	14.1 (4.5–23.7)	14.3 (4.3–24.4)	1.7 (1.6–1.9)	1.8 (1.7–1.8)
White	19.2 (18.3–20.2)	17.8 (16.9–18.7)	2.0 (1.9–2.0)	1.7 (1.6–1.7)
IMD				
IMD 1 (most deprived)	23.8 (21.5–26.0)	20.2 (18.2–22.3)	2.4 (2.3–2.4)	1.8 (1.8–1.8)
IMD 2	20.3 (18.1–22.4)	18.4 (16.4–20.4)	2.1 (2.0–2.1)	1.7 (1.7–1.7)
IMD 3	19.2 (17.3–21.1)	17.8 (16.0–19.7)	1.9 (1.8–1.9)	1.6 (1.6–1.6)
IMD 4	18.1 (16.3–19.9)	17.7 (15.8–19.5)	1.7 (1.7–1.8)	1.5 (1.5–1.5)
IMD 5 (least deprived)	16.1 (14.5–17.6)	16.0 (14.4–17.7)	1.6 (1.6–1.6)	1.4 (1.4–1.5)
Region				
East	19.5 (16.9–22.1)	18.7 (16.2–21.2)	2.0 (1.9–2.0)	1.7 (1.7–1.7)
East Midlands	18.3 (15.6–21.1)	17.1 (14.5–19.7)	1.8 (1.7–1.8)	1.5 (1.5–1.5)
London	18.3 (15.9–20.7)	16.8 (14.4–19.3)	1.9 (1.9–1.9)	1.7 (1.7–1.7)
North East	19.9 (15.9–24.0)	17.2 (13.4–21.1)	2.3 (2.2–2.4)	1.8 (1.8–1.8)
North West	20.4 (17.7–23.1)	18.6 (16.1–21.1)	2.1 (2.1–2.2)	1.7 (1.7–1.7)
South East	18.2 (16.2–20.2)	17.5 (15.5–19.5)	1.8 (1.7–1.8)	1.5 (1.5–1.6)
South West	19.2 (16.6–21.9)	18.4 (15.9–20.9)	1.9 (1.9–2.0)	1.6 (1.6–1.7)
West Midlands	20.7 (18.0–23.4)	19.0 (16.4–21.6)	1.7 (1.7–1.8)	1.4 (1.4–1.4)
Yorkshire and the Humber	20.7 (17.9–23.4)	18.4 (15.7–21.1)	2.0 (2–2.1)	1.6 (1.6–1.6)
Religion				
Religious affiliation	19.1 (18.0–20.3)	18.4 (17.1–19.6)	1.7 (1.7–1.8)	1.6 (1.6–1.6)
No religious affiliation	19.6 (18.4–20.9)	17.5 (16.4–18.7)	2.4 (2.4–2.5)	1.7 (1.7–1.7)
Sex				
Female	22.5 (21.4–23.7)	21.2 (20.0–22.3)	2.4 (2.4–2.5)	2.1 (2.1–2.1)
Male	15.9 (14.7–17.1)	14.9 (13.7–16.0)	1.4 (1.3–1.4)	1.2 (1.1–1.2)

CI, 95% confidence interval; IMD, Indices of Multiple Deprivation.

a This is the unadjusted model.

b Fully adjusted model; adjusted for age, country of birth, English as a first language, ethnicity, sex, IMD (quintile), region, religious affiliation, and disability status.

**Table 3.** Estimated proportion of individuals with a probable Common Mental Disorder (CMD) that receive treatment in Improving Access to Psychological Therapies (IAPT) broken down by socio-demographic characteristics

Exposure	Model 1^a^% (95% CI)	Model 2^b^% (95% CI)
Age		
18–24	14.2 (12.0–17.5)	13.4 (11.3–16.5)
25–34	12.8 (11.3–14.8)	12.4 (10.8–14.4)
35–44	11.6 (10.6–12.9)	11.2 (10.2–12.4)
45–54	8.9 (8.3–9.7)	8.8 (8.1–9.5)
55–64	7.3 (6.7–8.0)	7.3 (6.7–8.0)
65 and over	4.7 (4.3–5.1)	4.6 (4.3–5.1)
Country of birth		
Born outside the UK	7.2 (6.3–8.4)	7.1 (6.1–8.7)
Born within the UK	10.4 (9.9–10.9)	9.3 (8.8–9.8)
Disability		
Disabled	6.8 (6.4–7.3)	7.9 (7.4–8.5)
Not disabled	11.5 (10.8–12.1)	9.6 (9.1–10.2)
English as first language		
English first language	10.1 (9.7–10.6)	9.1 (8.7–9.6)
English not first language	6.9 (5.9–8.4)	7.8 (6.2–10.6)
Ethnicity		
Asian	7.2 (6.2–8.7)	6.6 (5.4–8.4)
Black	7.9 (6.1–11.5)	7.2 (5.5–10.5)
Mixed	9.7 (7.6–13.5)	6.9 (5.3–9.9)
Other ethnic group	12.5 (7.3–38.0)	12.6 (7.4–40.3)
White	10.2 (9.7–10.7)	9.3 (8.8–9.8)
IMD		
IMD 1 (most deprived)	10.0 (9.1–11.0)	9.1 (8.2–10.1)
IMD 2	10.2 (9.3–11.4)	9.2 (8.3–10.4)
IMD 3	9.7 (8.8–10.8)	8.9 (8.1–10.0)
IMD 4	9.7 (8.8–10.7)	8.6 (7.8–9.6)
IMD 5 (least deprived)	10.0 (9.1–11.1)	9.0 (8.2–10)
Region		
East	10.0 (8.9–11.6)	9.0 (7.9–10.4)
East Midlands	9.6 (8.3–11.3)	8.5 (7.4–10)
London	10.4 (9.2–11.9)	10.1 (8.8–11.8)
North East	11.6 (9.6–14.6)	10.4 (8.5–13.4)
North West	10.5 (9.3–12.1)	9.1 (8.0–10.5)
South East	9.6 (8.7–10.8)	8.8 (7.9–9.9)
South West	10.1 (8.9–11.7)	8.9 (7.9–10.3)
West Midlands	8.4 (7.4–9.6)	7.5 (6.6–8.7)
Yorkshire and the Humber	9.8 (8.6–11.3)	8.9 (7.7–10.4)
Religion		
Religious affiliation	9.1 (8.6–9.7)	8.5 (8.0–9.1)
No religious affiliation	12.4 (11.6–13.3)	9.8 (9.2–10.5)
Sex		
Female	10.8 (10.3–11.4)	10.1 (9.6–10.7)
Male	8.5 (7.9–9.2)	7.7 (7.2–8.4)

CI, 95% confidence interval; IMD, Indices of Multiple Deprivation.

a This is the unadjusted model.

b Fully adjusted model; adjusted for age, country of birth, English as a first language, ethnicity, sex, IMD (quintile), region, religious affiliation, and disability status.

**Table 4.** NS-SEC and Qualifications (25+ model) showing the proportions of people; with probable common mental disorder (CMD), accessing Improving Access to Psychological Therapies (IAPT) and the comparison

Adjustment	Variable	Level	Probability of having a CMD			Probability of access to IAPT			Probability of accessing IAPT amongst those with a probable CMD		
			Estimate	LCI	UCI	Estimate	LCI	UCI	Estimate	LCI	UCI
Unadjusted^a^	NS-SEC	Management & professional	16.2	15.0	17.4	1.6	1.6	1.6	9.9	9.1	10.7
		Intermediate	18.6	16.1	21.1	2.1	2.1	2.1	11.3	9.9	13.0
		Small employers & own account	14.1	11.5	16.6	1.1	1.1	1.2	8.0	6.7	9.8
		Lower supervisory & technical	17.9	14.6	21.1	1.4	1.4	1.5	8.0	6.8	9.8
		Semi-routine & routine	20.8	18.7	22.8	1.9	1.8	1.9	8.9	8.1	9.9
		Missing or not applicable	24.7	17.8	31.6	2.8	2.7	2.8	11.1	8.7	15.4
		Never worked or unemployed	26.9	22.5	31.3	2.3	2.3	2.4	8.7	7.4	10.4
	Qualifications	Below a degree level	19.1	17.8	20.4	2.0	2.0	2.0	10.3	9.7	11.1
		Degree or above a degree level	16.9	15.5	18.2	1.7	1.7	1.8	10.3	9.5	11.2
		No qualifications	20.1	17.0	23.3	1.3	1.2	1.3	6.3	5.4	7.4
Fully Adjusted^b^	NS-SEC	Management & professional	15.9	14.5	17.3	1.4	1.4	1.4	8.7	8.0	9.6
		Intermediate	16.6	14.3	19.0	1.6	1.6	1.6	9.6	8.4	11.2
		Small employers & own account	15.5	12.8	18.2	1.2	1.2	1.2	7.9	6.7	9.6
		Lower supervisory & technical	17.2	14.0	20.4	1.4	1.4	1.4	8.0	6.8	9.9
		Semi-routine & routine	17.9	15.9	19.8	1.6	1.6	1.6	8.9	8.0	10.0
		NS-SEC missing or not applicable	19.5	12.9	26.2	1.6	1.6	1.6	8.2	6.1	12.5
		Never worked or unemployed	20.9	16.7	25.1	1.7	1.6	1.7	7.9	6.6	9.9
	Qualifications	Below a degree level	16.8	15.6	18.0	1.5	1.5	1.5	9.1	8.5	9.9
		Degree or above a degree level	16.3	14.8	17.8	1.5	1.5	1.5	9.4	8.6	10.3
		No qualifications	19.2	15.7	22.7	1.3	1.2	1.3	6.5	5.5	8.0

NS-SEC, National Statistics Socio-Economic Classification; LCI, Lower Confidence Interval; UCI, Upper Confidence Interval.

a Unadjusted model.

b Fully adjusted model; adjusted for age, country of birth, English as a first language, ethnicity, sex, Index of Multiple Deprivation (quintiles), region, religious affiliation, disability status, qualifications and NS-SEC.

#### Age and sex

Adults aged 25- to 34-years were most likely to have a probable CMD [23.2% (95% CI 20.1–26.3%)) and this declined with age. Similarly, among all individuals in IAPT, treatment rates decreased with age with only 0.7% (95% CI 0.6–0.7%) of adults aged 65- to 100-years old receiving treatment. Among those with probable CMDs, 18- to 24-year-olds were most likely to access IAPT [14.2% (95% CI 12.0–17.5%)] and adults 65 and over least likely to access IAPT [4.7% (95% CI 4.3–5.1%)]. Adjusting for other factors had little effect on the estimated differences in access to IAPT by age and sex.

Males were less likely to have a probable CMD [15.9% (95% CI 14.7–17.1%)] and to receive treatment through IAPT [1.4% (95% CI 1.3–1.4%)]. There is a statistically significant underrepresentation of males in IAPT with only 8.5% (95% CI 7.9–9.2%) of males with CMD entering this service compared to 10.8% (95% CI 10.3–11.4%) of females. When adjusting for other factors the difference reduced slightly but remained significant.

#### Ethnicity, country of birth, English as a first language and religion

The Mixed ethnic group had the highest proportion of probable CMDs [30.4% (95% CI 21.9–38.8%)] when comparing against the other ethnic groups (range 14.1 to 21.8%). They were also most likely to be receiving treatment in IAPT [2.9% (95% CI 2.8–3.1%)]. When estimating the proportion of individuals with CMD who received treatment in IAPT, 9.7% (95% CI 7.6–13.5%) of the Mixed ethnic group received treatment compared to 10.2% (95% CI 9.7–10.7%) of the White ethnic group. The Asian ethnic group [7.2% (95% CI 6.2–8.7%)] were significantly underrepresented in IAPT when compared to the White ethnic group. Adjusting for other factors reduced the differences, but the Asian ethnic group remained significantly underrepresented in IAPT compared to the White ethnic group.

Those born in the UK were (non-significantly) more likely to have a probable CMD over those born outside of the UK [19.6% (95% CI 18.7–20.6%) compared to 17.0% (95% CI 14.6–19.4%)]. However, those born outside the UK were significantly less likely to receive treatment in IAPT [1.2% (95% CI 1.2–1.3%)] compared to 2.0% (95% CI 2.0–2.0%) and consequently only 7.2% (95% CI 6.3–8.4%) of those with probable CMD received treatment in IAPT compared with 10.4% (95%CI 9.9–10.9%) of UK born patients. This difference reduced but remained significant when adjusted for other factors.

CMDs affected 19.6% (95% CI 18.7–20.5%) of people whose first language was English compared to 15.4% (95% CI 12.7–18.1%) of people for who English was not their first language. Also, there was a higher proportion of patients receiving treatment in IAPT where English was their first language [2.0% (95% CI 2.0–2.0%) compared to 1.1% (95% CI 1.0–1.1%)] where English was not their first language. When comparing there was a significant difference in representation with those where English is not the first language being underrepresented [6.9% (95% CI 5.9–8.4%)] compared to those where English was the first language [10.1% (95% CI 9.7–10.6%)]. Adjusting for other factors made this difference non-significant.

There was no difference in the proportion of people with a probable CMD by religious affiliation [no religion: 19.6% (95% CI 18.4–20.9%); any religion affiliation: 19.1% (95% CI 18.0–20.3%)]. People with no religion were significantly more likely to be treated in IAPT [2.4% (95% CI 2.4–2.5%) compared to 1.7% (95% CI 1.7–1.8%)]. Those with a religious affiliation and probable CMD were significantly underrepresented in IAPT [9.1% (95% CI 8.6–9.7%) compared with 12.4% (95% CI 11.6–13.3%)]. Adjusting for other factors reduced the difference, but having a religious affiliation remained significantly associated with being underrepresented in IAPT.

#### Disability status

Disabled people had higher levels of probable CMD [31.6% (95% CI 29.5–33.8%)] compared to those who were not disabled [16.4% (95%CI 15.5–17.3%)] and there was a higher proportion of disabled people receiving treatment in IAPT [2.2% (95% CI 2.1–2.2%) compared to 1.9% (95% CI 1.9–1.9%)]. However, only 6.8% (95% CI 6.4–7.3%) of disabled people with a probable CMD are being treated in IAPT compared with 11.5% (95%CI 10.8–12.1%) of non-disabled people, indicating disabled people are significantly underrepresented in IAPT. After adjustment, these differences were attenuated but remained significant, suggesting having a disability is negatively associated with access to IAPT.

#### Highest qualification (sample age 25+)

Having no academic or professional qualifications was associated with a higher level of probable CMDs [20.1% (95% CI 17.0–23.3%)] compared to below a degree qualification [19.1% (95% CI 17.8–20.4%)] and degree level and above [16.9% (95%CI 15.5–18.2%)] (Table 4). Despite this, the no qualifications group were least likely to be treated in IAPT [1.3% (95% CI 1.2–1.3%)] and this was significantly different to those with any other qualifications [below a degree level; 2.0% (95% CI 2.0–2.0%), degree level and above; 1.7% (95% CI 1.7–1.8%)]. They were also significantly underrepresented in IAPT as a proportion of those with probable CMD [6.3% (95% CI 5.4–7.4%)] compared to 10.3% (95% CI 9.7–11.1%) for those with a below a degree level qualification and 10.3% (95% CI 9.5–11.2%) of those with a degree level and above. After adjustment, this difference reduced but remained significant.

### Additional analyses

Further analyses on local area deprivation, region and NS-SEC can be found in Tables 2, 3 and 4. There was little difference in IAPT treatment rates among those with a probable CMD by IMD (range 9.7–10.2%) with those in IMD category 2 (1 being most deprived) most represented in IAPT [IMD 2: 10.2% (95% CI 9.3–11.4)]. The biggest difference between regions was between the West Midlands [8.4% (95% CI 7.4–9.6)] and the North East [11.6% (95% CI 9.6–14.6)].

## Discussion

This study found that older adults, males, people born outside of the UK, people with a religious affiliation, people from Asian ethnic backgrounds, people with a disability and those without any academic or professional qualifications appear underrepresented in IAPT as a proportion of those with a probable CMD.

Research acknowledges that older adults are underrepresented in mental health treatment (Department of Health, 2001), including IAPT (Clark, 2018) despite evidence that they may have better outcomes in IAPT than working age adults (NHS Digital, 2022; Saunders et al., 2021), and this is supported by the results of this study. Having a disability was also associated with reduced access to IAPT. The prevalence of CMDs is higher in those with a disability and this supports previous research demonstrating that people with disabilities are at a higher risk of experiencing poor mental health (NHS England, 2016), although it is important to note that for many individuals' mental ill-health itself can be classified as a disability. The IAPT programme has expanded to offer integrated services which provide treatment for CMDs in individuals with long term physical health conditions, which go some way to addressing the treatment gap for those with disabilities (Clark, 2018).Targeted programmes within IAPT which help those with long term conditions have been effective at reducing depression and anxiety (Toffolutti et al., 2021).

Men are less likely to seek support than women for their mental health (Bogdanova, Cooper, Ahmad, McManus, & Shoham, 2022) despite evidence that men are much more likely to take their own life (Office for National Statistics, 2021; Vogel, Wade, & Haake, 2006). The differences in access in IAPT are consistent with this existing research. Increasing engagement with men may help to reduce poorer outcomes. This outreach should be targeted to ensure there are greater numbers of referrals for men, and barriers need to be broken down to encourage men to seek this support when needed.

Research reports that marginalised groups are at higher risk of poorer mental health (Nyashanu, Ganga, & Chenneville, 2022) as they are more likely to experience downward social mobility due to racism, discrimination, language barriers or lack of cultural knowledge (Das-Munshi et al., 2012). Studies also report that mental health services continue to be inaccessible for these groups (Das-Munshi et al., 2012). Research has found that individuals from ethnic minority backgrounds are less likely to consult GPs for CMDs and when they do, they are less likely to be diagnosed (Bogdanova et al., 2022; Cooper et al., 2013). Evidence suggests that minority groups are more likely to be referred to IAPT via secondary care routes and are less likely to self-refer to the service, showing differences in health seeking behaviours (Harwood et al., 2021). Recent research using the 2007 and 2014 APMS data also shows treatment inequalities are widening over time by ethnic group (Ahmad et al., 2022).

There was a significant underrepresentation of those born outside of the UK compared to those born in the UK in IAPT, confirming previous research suggesting that migrants are likely to be disadvantaged regarding access to support for mental health issues (Bhavsar et al., 2021; Das-Munshi et al., 2012). Despite this, not having English as a first language was not identified as a significant barrier to accessing IAPT services. Given research has indicated language barriers can impact access to mental health treatment (Scheppers, van Dongen, Dekker, Geertzen, & Dekker, 2006), this finding may highlight some positive actions taken by IAPT services to increase accessibility. For example, the IAPT manual has several initiatives such as bilingual clinicians and producing leaflets and information in community languages (Harwood et al., 2021) which may partly explain this finding. For ethnic minority groups, migrants and those where English is not their first language further research is needed to understand the barriers to treatment that these groups face, which might inform policies to improve access for these groups.

Religion can be an important factor affecting attitudes to accessing support for mental health (Loewenthal et al., 2012). Religious people often feel that the significance of their beliefs are not understood by mental health professionals (McCullough & Larson, 1999), despite evidence that religious and faith-based adaptions to psychological therapy might improve outcomes (Arundell, Barnett, Buckman, Saunders, & Pilling, 2021). Some groups and communities still stigmatise mental illness, seeing it as a punishment and fearing discrimination from their community, meaning they may opt for spiritual treatments (Yu, Yang, Yu, & Liu, 2022). Despite research suggesting that religious involvement could provide a sense of belonging and spirituality and consequently benefits to mental health (King et al., 2006; McCullough & Larson, 1999), in this study, there was no difference in the proportion of people with CMDs between those with and without religious affiliation.

Previous research has identified complex relationships between school experiences, educational qualifications and mental health problems (Esch et al., 2014; Fergusson, McLeod, & Horwood, 2015).This is reflected in the proportion of CMDs experienced by those without any academic and professional qualifications, yet this group were also less represented in IAPT treatment. There is little research into the potential barriers in access for individuals with lower educational attainment but efforts to better understand such factors might help services improve access for these individuals.

Lastly, although the self-referral model has been designed to improve the overall access to IAPT, studies have found that the self-referral process can be overwhelming and act as a barrier in access for certain socio-demographics (Thomas et al., 2020). Individuals who are aware of mental health services in England, have access to internet-enabled devices and are confident in using these devices could be more likely to self-refer than others. Therefore, it could be argued the self-referral option could lead to increased inequalities in access between groups.

### Strengths and limitations

This study benefitted from a unique dataset linking the 2011 Census to national IAPT data. Unlike studies solely based on sample frames, we generated high-quality population-based information with large sample size and high data coverage. Data collected by UKHLS provided large representative sample of the English population with a probable CMD which increased validity of the findings. This study used a validated and reliable measure to identify probable CMDs.

The 2017/18 IAPT dataset was used for the analysis, and therefore findings may not reflect the current representation in IAPT which showed relative increases in referrals that started treatment across all socio-demographic groups (NHS Digital, 2022). Similarly, some 2011 Census variables particularly qualifications may have changed in between the census and IAPT access. Future research should look at later years of data to compare changes in IAPT access over time.

Despite GHQ-12 being a reliable and commonly used measure, previous studies have questioned validity of GHQ-12 for identifying CMDs (Donath, 2001). We used GHQ-12 as a proxy of CMDs, but not all individuals scoring 4+ are likely to require support for a CMD. It is also possible that people with serious mental illness, who should be referred to secondary mental health services rather than IAPT, might score 4+ on GHQ-12. Conversely, GHQ-12 might not capture well some conditions, such as obsessive-compulsive disorder and post-traumatic stress disorder, that are covered by IAPT. We also do not account for other services or potential treatments for CMDs like private care or medication which may reduce the need for IAPT. For that reason, we can only assess the uptake from IAPT, it is not possible to say if groups underrepresented in IAPT are more likely to receive other types of treatment instead. Further research combining different data sources to assess different treatments for CMDs combined would be desirable. Additionally, the anticipated access rates for the IAPT programme are typically based on results from the Adult Psychiatric Morbidity Survey (APMS) which uses the Clinical Interview Schedule Revised (CIS-R) to determine CMD prevalence rates in England which could result in discrepancies with the UKHLS.

Lastly, we only included individuals seen at least once in IAPT, meaning we evaluated the initial access to mental health services as an unmet need. This does not consider the quality and efficacy of the care they receive. Retention of patients, treatment length and outcomes should be evaluated to fully understand socio-demographic differences in access to mental health services. Due to data availability, we could not include or control for clinical severity scores, so future iterations of this study should incorporate these to provide a more accurate representation of people with probable CMD in IAPT.

## Conclusions

This study aimed to identify the representativeness of the IAPT population amongst people with probable CMDs. The study found underrepresentation of several key groups including older adults, males, ethnic minority groups, those born outside of the UK and those with no academic or professional qualifications. Whilst these results are not necessarily unique to IAPT in the context of mental healthcare, they should inform future expansion of IAPT services targeting these groups to ensure they get equal treatment of their CMD. Future research should consider repeating the study with later years of IAPT and 2021 Census data, and consider to further explore the measure for probable CMD prevalence by comparing GHQ-12 to the more detailed CIS-R based on the next APMS survey. To address knowledge gaps, future research should also incorporate analysis looking at differences in treatment length, dropout rates and outcomes of patients who completed minimal treatment in IAPT. This will provide more accurate representation of people with CMDs in IAPT and greater understanding of the unmet need. Research should also incorporate patients' perspective of barriers to access to mental health services.

## Supporting information

Sharland et al. supplementary material 1 Sharland et al. supplementary material

Sharland et al. supplementary material 2 Sharland et al. supplementary material

Sharland et al. supplementary material 3 Sharland et al. supplementary material

## Supplementary material

The supplementary material for this article can be found at https://doi.org/10.1017/S0033291723001010

## References

[ref1] Ahmad, G., McManus, S., Cooper, C., Hatch, S. L., & Das-Munshi, J. (2022). Prevalence of common mental disorders and treatment receipt for people from ethnic minority backgrounds in England: Repeated cross-sectional surveys of the general population in 2007 and 2014. British Journal of Psychiatry, 221(3), 520–527. 10.1192/bjp.2021.179. PMC7616892 35049474

[ref2] Arundell, L.-L., Barnett, P., Buckman, J. E. J., Saunders, R., & Pilling, S. (2021). The effectiveness of adapted psychological interventions for people from ethnic minority groups: A systematic review and conceptual typology. Clinical Psychology Review, 88, 102063. 10.1016/j.cpr.2021.102063. 34265501 PMC8591374

[ref3] Bhavsar, V., Jannesari, S., McGuire, P., MacCabe, J. H., Das-Munshi, J., Bhugra, D., … Hatch, S. L. (2021). The association of migration and ethnicity with use of the improving access to psychological treatment (IAPT) programme: A general population cohort study. Social Psychiatry and Psychiatric Epidemiology: The International Journal for Research in Social and Genetic Epidemiology and Mental Health Services, 56(11), 1943–1956. 10.1007/s00127-021-02035-7. PMC8519879 33591376

[ref4] Bogdanova, N., Cooper, C., Ahmad, G., McManus, S., & Shoham, N. (2022). Associations between sociodemographic characteristics and receipt of professional diagnosis in common mental disorder: Results from the adult psychiatric morbidity survey 2014. Journal of Affective Disorders, 319, 112–118. 10.1016/j.jad.2022.09.085. 36155230

[ref5] Brown, S., Harris, M. N., Srivastava, P., & Taylor, K. B. (2018). Mental health and reporting bias: Analysis of the GHQ-12. In SSRN Electronic Journal, IZA Discussion Paper No. 11771, 1–40. 10.2139/ssrn.3249885.

[ref6] Buckland, S. T. (1984). Monte Carlo confidence intervals. Biometrics, 40(3), 811–817. 10.2307/2530926.

[ref7] Clark, D. M. (2018). Realizing the mass public benefit of evidence-based psychological therapies: The IAPT program. Annual Review of Clinical Psychology, 14, 159–183. 10.1146/annurev-clinpsy-050817. PMC5942544 29350997

[ref8] Cooper, C., Spiers, N., Livingston, G., Jenkins, R., Meltzer, H., Brugha, T., … Bebbington, P. (2013). Ethnic inequalities in the use of health services for common mental disorders in England. Social Psychiatry and Psychiatric Epidemiology, 48(5), 685–692. 10.1007/s00127-012-0565-y. 22893107

[ref9] Das-Munshi, J., Leavey, G., Stansfeld, S. A., & Prince, M. J. (2012). Migration, social mobility and common mental disorders: Critical review of the literature and meta-analysis. Ethnicity & Health, 17(1–2), 17–53. 10.1080/13557858.2011.632816. 22074468

[ref10] Department of Health. (2001). National Service Framework for Older People. Retrieved August 10, 2022, from https://www.gov.uk/government/publications/quality-standards-for-care-services-for-older-people.

[ref11] Dixon-Woods, M., Kirk, D., Agarwal, S., Annandale, E., Arthur, T., Harvey, J., … Sutton, A. (2005). Vulnerable groups and access to health care: A critical interpretive review. Report for the national co-ordinating centre for NHS service delivery and organisation R & D *(*NCCSDO*)*. London: NCCSDO. http://www.sdo.nihr.ac.uk/files/project/SDO_ES_08-1210-025_V01.pdf.

[ref12] Donath, S. (2001). The validity of the 12-item general health questionnaire in Australia: A comparison between three scoring methods. Australian & New Zealand Journal of Psychiatry, 35(2), 231–235. 10.1046/j.1440-1614.2001.00869.x. 11284906

[ref13] Endsley, P., Weobong, B., & Nadkarni, A. (2017). The psychometric properties of GHQ for detecting common mental disorder among community dwelling men in Goa, India. Asian Journal of Psychiatry, 28(2017), 106–110. 10.1016/j.ajp.2017.03.023. 28784361 PMC5565797

[ref14] Esch, P., Bocquet, V., Pull, C., Couffignal, S., Lehnert, T., Graas, M., … Ansseau, M. (2014). The downward spiral of mental disorders and educational attainment: A systematic review on early school leaving. BMC Psychiatry, 14(1), 1–13. 10.1186/s12888-014-0237-4. PMC4244046 25159271

[ref15] Fergusson, D. M., McLeod, G. F. H., & Horwood, L. J. (2015). Leaving school without qualifications and mental health problems to age 30. Social Psychiatry and Psychiatric Epidemiology, 50(3), 469–478. 10.1007/s00127-014-0971-4. 25314915

[ref16] Fone, D., White, J., Farewell, D., Kelly, M., John, G., Lloyd, K., … Dunstan, F. (2014). Effect of neighbourhood deprivation and social cohesion on mental health inequality: A multilevel population-based longitudinal study. Psychological Medicine, 44(11), 2449–2460. 10.1017/S0033291713003255. 24451050

[ref17] Fryers, T., Melzer, D., Jenkins, R., & Brugha, T. (2005). The distribution of the common mental disorders: Social inequalities in Europe. Clinical Practice and Epidemiology in Mental Health, 1(14), 1–12. 10.1186/1745-0179-1-14. 16143042 PMC1242241

[ref18] Goldberg, D. P., Gater, R., Sartorius, N., Ustun, T. B., Piccinelli, M., Gureje, O., & Rutter, C. (1997). The validity of two versions of the GHQ in the WHO study of mental illness in general health care. Psychological Medicine, 27(1), 191–197. 10.1017/S0033291796004242. 9122299

[ref19] Harris, K. M., Edlund, M. J., & Larson, S. L. (2006). Religious involvement and the use of mental health care. Health Services Research, 41(2), 395–410. 10.1111/j.1475-6773.2006.00500.x. 16584455 PMC1702510

[ref20] Harwood, H., Rhead, R., Chui, Z., Bakolis, I., Connor, L., Gazard, B., … Hatch, S. L. (2021). Variations by ethnicity in referral and treatment pathways for IAPT service users in South London. Psychological Medicine, 53(3), 1084–1095. 10.1017/S0033291721002518. 34334151 PMC9976018

[ref21] John, A., Saunders, R., Desai, R., Bell, G., Fearn, C., Buckman, J. E. J., … Stott, J. (2022). Associations between psychological therapy outcomes for depression and incidence of dementia. Psychological Medicine, FirstView, 1–11. 10.1017/S0033291722002537 PMC10476047 36106698

[ref22] Kaminska, O., & Lynn, P. (2019). Weighting and Sample Representation: Frequently Asked Questions. Retrieved June 1, 2022, from https://www.understandingsociety.ac.uk/sites/default/files/downloads/documentation/user-guides/mainstage/weighting_faqs.pdf.

[ref23] King, M., Weich, S., Nazroo, J., & Blizard, B. (2006). Religion, mental health and ethnicity. EMPIRIC – A national survey of England. Journal of Mental Health, 15(2), 153–162. 10.1080/09638230600608891.

[ref24] Loewenthal, D., Mohamed, A., Mukhopadhyay, S., Ganesh, K., & Thomas, R. (2012). Reducing the barriers to accessing psychological therapies for Bengali, Urdu, Tamil and Somali communities in the UK: Some implications for training, policy and practice. British Journal of Guidance & Counselling, 40(1), 43–66. 10.1080/03069885.2011.621519.

[ref25] McCullough, M. E., & Larson, D. B. (1999). Religion and depression: A review of the literature. Twin Research, 2(2), 126–136. 10.1375/twin.2.2.126. 10480747

[ref26] McManus, S., Bebbington, P., Jenkins, R., & Brugha, T. (Eds.) (2016). Mental health and wellbeing in England: Adult psychiatric morbidity survey 2014. Leeds: NHS Digital. Retrieved from apms-2014-full-rpt.pdf (publishing.service.gov.uk).

[ref27] Mercer, S. W., & Watt, G. C. M. (2007). The inverse care law: Clinical primary care encounters in deprived and affluent areas of Scotland. Annals of Family Medicine, 5(6), 503–510. 10.1370/afm.778. 18025487 PMC2094031

[ref28] Naylor, C., Parsonage, M., Mcdaid, D., Knapp, M., Fossey, M., & Galea, A. (2012). Long-term condition and mental health: The cost of comorbidities. The King’s Fund and Centre for Mental Health. https://www.centreformentalhealth.org.uk/sites/default/files/2018-09/Cost_of_Comorbidities.pdf.

[ref29] NHS Digital. (2018). *Psychological Therapies: Annual report on the use of IAPT*. *Data Quality Report* 2017–2018. Retrieved from https://files.digital.nhs.uk/5F/5732D6/psych-ther-ann-2017-18-dq-rep.xls.

[ref30] NHS Digital. (2022). *Psychological Therapies*, *Annual report on the use of IAPT services* 2021*/*22. Retrieved from https://digital.nhs.uk/data-and-information/publications/statistical/psychological-therapies-annual-reports-on-the-use-of-iapt-services/annual-report-2021-22.

[ref31] NHS England. (2016). *The Five Year Forward View for Mental Health: A report from the independent mental health taskforce for the NHS in England*. Retrieved from https://www.england.nhs.uk/wp-content/uploads/2016/02/Mental-Health-Taskforce-FYFV-final.pdf.

[ref32] NHS England. (2019). *NHS Mental Health Implementation Plan* 2019*/*20–2023*/*24. Retrieved from https://www.longtermplan.nhs.uk/wp-content/uploads/2019/07/nhs-mental-health-implementation-plan-2019-20-2023-24.pdf.

[ref33] Nunes, M. A., Pinheiro, A. P., Bessel, M., Brunoni, A. R., Kemp, A. H., Benseñor, I. M., … Schmidt, M. I. (2016). Common mental disorders and sociodemographic characteristics: Baseline findings of the Brazilian Longitudinal Study of Adult Health (ELSA-Brasil). Brazilian Journal of Psychiatry, 38(2), 91–97. 10.1590/1516-4446-2015-1714. 27304755 PMC7111374

[ref34] Nyashanu, M., Ganga, G., & Chenneville, T. (2022). Exploring the impact of religion, superstition, and professional cultural competence on access to HIV and mental health treatment among Black Sub-Sahara African Communities in the English City of Birmingham. Journal of Religion and Health, 61(1), 252–268. 10.1007/s10943-021-01298-3. 34085190

[ref35] Office for National Statistics. (2021). Suicides in England and Wales: 2020 Registrations. Retrieved June 24, 2022, from https://www.ons.gov.uk/peoplepopulationandcommunity/birthsdeathsandmarriages/deaths/bulletins/suicidesintheunitedkingdom/2020registrations.

[ref36] Office for National Statistics. (2022). About the census. Retrieved November 16, 2022, from https://www.ons.gov.uk/census/aboutcensus/aboutthecensus.

[ref37] Saunders, R., Buckman, J. E. J., Stott, J., Leibowitz, J., Aguirre, E., John, A., … Pilling, S. (2021). Older adults respond better to psychological therapy than working-age adults: Evidence from a large sample of mental health service attendees. Journal of Affective Disorders, 294(June), 85–93. 10.1016/j.jad.2021.06.084. 34274792 PMC8411661

[ref38] Scheppers, E., van Dongen, E., Dekker, J., Geertzen, J., & Dekker, J. (2006). Potential barriers to the use of health services among ethnic minorities: A review. Family Practice, 23(3), 325–348. 10.1093/fampra/cmi113. 16476700

[ref39] Simpson, S. M., Krishnan, L. L., Kunik, M. E., & Ruiz, P. (2007). Racial disparities in diagnosis and treatment of depression: A literature review. The Psychiatric Quarterly, 78(1), 3–14. 10.1007/s11126-006-9022-y. 17102936

[ref40] Smyth, N., Buckman, J. E. J., Naqvi, S. A., Aguirre, E., Cardoso, A., Pilling, S., … Saunders, R. (2022). Understanding differences in mental health service use by men: An intersectional analysis of routine data. Social Psychiatry and Psychiatric Epidemiology, 57, 2065–2077. 10.1007/s00127-022-02256-4. 35318495 PMC9477949

[ref41] Stochl, J., Böhnke, J. R., Pickett, K. E., & Croudace, T. J. (2016). An evaluation of computerized adaptive testing for general psychological distress: Combining GHQ-12 and Affectometer-2 in an item bank for public mental health research. BMC Medical Research Methodology, 16(1), 1–15. 10.1186/s12874-016-0158-7. 27206714 PMC4875708

[ref42] Thomas, F., Hansford, L., Ford, J., Wyatt, K., McCabe, R., & Byng, R. (2020). How accessible and acceptable are current GP referral mechanisms for IAPT for low-income patients? Lay and primary care perspectives. Journal of Mental Health, 29(6), 706–711. 10.1080/09638237.2019.1677876. 31682539

[ref43] Toffolutti, V., Stuckler, D., McKee, M., Wolsey, I., Chapman, J., J Pimm, T., … M Clark, D. (2021). The employment and mental health impact of integrated improving access to psychological therapies: Evidence on secondary health care utilization from a pragmatic trial in three English counties. Journal of Health Services Research and Policy, 26(4), 224–233. 10.1177/1355819621997493. 33771070

[ref44] University of Essex: Institute for Social and Economic Research. (2022). Understanding society: Waves 1–11, 2009–2020 and harmonised BHPS: Waves 1–18, 1991–2009. [data collection] (15th ed.). Essex: UK Data Service. 10.5255/UKDA-SN-6614-16.

[ref45] Van Voorhees, B. W., Walters, A. E., Prochaska, M., & Quinn, M. T. (2007). Reducing health disparities in depressive disorders outcomes between non-Hispanic Whites and ethnic minorities: A call for pragmatic strategies over the life course. Medical Care Research and Review: MCRR, 64(5 Suppl), 157S–194S. 10.1177/1077558707305424. 17766647

[ref46] Vogel, D. L., Wade, N. G., & Haake, S. (2006). Measuring the self-stigma associated with seeking psychological help. Journal of Counseling Psychology, 53(3), 325–337. 10.1037/0022-0167.53.3.325.

[ref47] Weich, S., Nazareth, I., Morgan, L., & King, M. (2007). Treatment of depression in primary care: Socio-economic status, clinical need and receipt of treatment. British Journal of Psychiatry, 191(2), 164–169. 10.1192/bjp.bp.106.032219. 17666502

[ref48] Yu, H., Yang, C.-C., Yu, P., & Liu, K. (2022). Emotion diffusion effect: Negative sentiment COVID-19 tweets of public organizations attract more responses from followers. PLOS ONE, 17(3), e0264794. 10.1371/journal.pone.0264794. 35259181 PMC8903302

