# Supplementary figures and images for: Socio-demographic differences in access to psychological treatment services: evidence from a national cohort study
Source: Psychol Med. 2023 May 17;53(15):7395–406. doi: 10.1017/S0033291723001010 (PMC10721408; doi:10.1017/S0033291723001010)

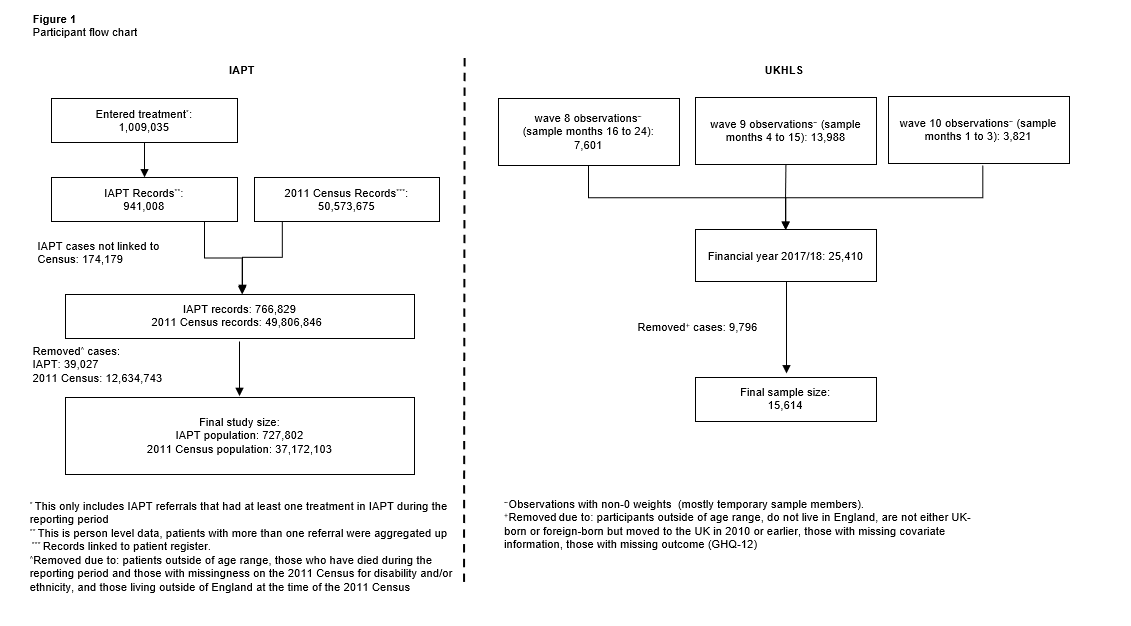

Supplement: Sharland et al. supplementary material 1 — Sharland et al. supplementary material [file S0033291723001010sup001.png]
