# Supplementary material for: Socio-demographic differences in access to psychological treatment services: evidence from a national cohort study
Source: Psychol Med. 2023 May 17;53(15):7395–406. doi: 10.1017/S0033291723001010 (PMC10721408; doi:10.1017/S0033291723001010)
Supplement: Sharland et al. supplementary material 2 — Sharland et al. supplementary material [file S0033291723001010sup002.docx]

**Supplementary Table 1**: List of variables used within the study

| **Variable** | **Categories** |
| --- | --- |
| Age | 18-24, 25-34, 35-44, 45-54, 55-64, 65+ |
| APPTYPE | Appointment type variable in IAPT.  This variable refers to the type of IAPT appointment participants received during each session. Participants were included if their first appointment type was coded as either treatment, assessment and treatment or review and treatment. |
| Country of birth | Born in the UK, Born outside of the UK |
| Disability Status | IAPT: Not disabled (not limited at all), disabled (limited a little or a lot). UKHLS Not disabled (No long-term health condition or disability; or: Has a long-term health condition or disability but no impairment. Disabled: Has a long-term health condition or disability and (at least one substantial impairment due to it; or is a proxy interview) |
| English as a first language | English first language, English not a first language |
| Ethnicity | Asian, Black, Mixed, Other ethnic group, White |
| Index of Multiple Deprivation (IMD) | Quintile (1: most deprived - 5: least deprived) |
| National Statistics Socio-Economic Classification (NS-SEC) | Management and professional, Intermediate, Small employers and own account, Lower supervisory and technical, Semi-routine and routine, Missing or not applicable, Never worked or long-term unemployment |
| Qualifications | Below a degree level, Degree level or above, No academic or professional qualification |
| Region | East, East Midlands, London, North East, North West, South East, South West, West Midlands, Yorkshire and the Humber |
| Religious affiliation | Religious affiliation, No religious affiliation |
| Sex | Female, Male |
