# Supplementary material for: Socio-demographic differences in access to psychological treatment services: evidence from a national cohort study
Source: Psychol Med. 2023 May 17;53(15):7395–406. doi: 10.1017/S0033291723001010 (PMC10721408; doi:10.1017/S0033291723001010)
Supplement: Sharland et al. supplementary material 3 — Sharland et al. supplementary material [file S0033291723001010sup003.docx]

**Supplementary Table 2:** Socio-demographic characteristics of the study population by outcome measure

| **Variable** | **Level** | | **IAPT** | | **Non-IAPT (Census population minus those in IAPT)** | | **UKHLS (GHQ 4+)** | | **UKHLS (GHQ <4)** | |
| --- | --- | --- | --- | --- | --- | --- | --- | --- | --- | --- |
|  |  |  | **Count** | **%** | **Count** | **%** | **Count** | **weighted %** | **Count** | **weighted %** |
| Age | | 18-24 | 131,475 | 18.1 | 3,909,253 | 10.5 | 128 | 10.71 | 443 | 8.4 |
|  |  | 25-34 | 172,993 | 23.8 | 5,661,842 | 15.2 | 282 | 16.81 | 1,013 | 12.9 |
|  |  | 35-44 | 143,701 | 19.7 | 5,922,776 | 15.9 | 481 | 16.11 | 1,939 | 15.1 |
|  |  | 45-54 | 134,523 | 18.5 | 6,886,922 | 18.5 | 660 | 19.67 | 2,542 | 17.7 |
|  |  | 55-64 | 86,534 | 11.9 | 5,864,106 | 15.8 | 600 | 17.56 | 2,568 | 17.8 |
|  |  | 65-100 | 58,576 | 8.0 | 8,927,204 | 24 | 673 | 19.14 | 4,285 | 28.2 |
| Country of Birth | | Born outside the UK | 66,211 | 9.1 | 5,336,492 | 14.4 | 337 | 8.83 | 1,590 | 10.4 |
|  |  | Born within the UK | 661,591 | 90.9 | 31,835,611 | 85.6 | 2,487 | 91.17 | 11,200 | 89.6 |
| Disability Status | | Not disabled | 595,304 | 81.8 | 31,156,019 | 83.8 | 1,878 | 67.91 | 10,568 | 82.8 |
|  |  | Disabled | 132,498 | 18.2 | 6,016,084 | 16.2 | 946 | 32.09 | 2,222 | 17.2 |
| English as a first Language | | English is the first language | 696,927 | 95.8 | 34,333,126 | 92.4 | 2,612 | 95.28 | 11,768 | 93.7 |
|  |  | English is not the first language | 30,875 | 4.2 | 2,838,977 | 7.6 | 212 | 4.72 | 1,022 | 6.3 |
| Ethnicity | | Asian | 36,736 | 5.0 | 2,717,595 | 7.3 | 244 | 4.73 | 1,037 | 5.1 |
|  |  | Black | 19,156 | 2.6 | 1,088,652 | 2.9 | 91 | 2.13 | 414 | 2.1 |
|  |  | Mixed | 16,936 | 2.3 | 558,931 | 1.5 | 73 | 2.02 | 179 | 1.2 |
|  |  | Other ethnic group | 5,169 | 0.7 | 288,833 | 0.8 | 16 | 0.34 | 71 | 0.5 |
|  |  | White | 649,805 | 89.3 | 32,518,092 | 87.5 | 2,400 | 90.78 | 11,089 | 91.1 |
| IMD (quintile) | | 1 - most deprived | 165,373 | 22.7 | 6,837,820 | 18.4 | 619 | 22.36 | 2,093 | 17.3 |
|  |  | 2 | 155,510 | 21.4 | 7,335,715 | 19.7 | 576 | 20.18 | 2,341 | 18.6 |
|  |  | 3 | 144,732 | 19.9 | 7,603,223 | 20.5 | 549 | 21.56 | 2,635 | 20.7 |
|  |  | 4 | 136,221 | 18.7 | 7,673,825 | 20.6 | 554 | 19.01 | 2,785 | 21.3 |
|  |  | 5 - least deprived | 125,966 | 17.3 | 7,721,520 | 20.8 | 526 | 16.89 | 2,936 | 22.1 |
| NS-SEC* | | 1. Management and professional | 180,456 | 30.3 | 11,119,953 | 33.4 | 888 | 33.91 | 4,722 | 39.8 |
|  |  | 2. Intermediate | 100,345 | 16.8 | 4,671,088 | 14 | 345 | 13.44 | 1,575 | 12.9 |
|  |  | 3. Small employers and own account | 37,713 | 6.3 | 3,313,288 | 10 | 164 | 6.45 | 972 | 8.9 |
|  |  | 4. Lower supervisory and technical | 35,222 | 5.9 | 2,419,932 | 7.3 | 144 | 7.28 | 727 | 7.1 |
|  |  | 5. Semi-routine and routine | 164,695 | 27.6 | 8,715,212 | 26.2 | 654 | 28.65 | 2,641 | 24.6 |
|  |  | Never worked or long-term unemployment | 36,728 | 6.2 | 1,720,732 | 5.2 | 128 | 5.69 | 401 | 3.7 |
|  |  | Missing or not applicable | 41,168 | 6.9 | 1,302,645 | 3.9 | 66 | 4.58 | 242 | 3.1 |
| Qualifications* | | Below a degree level | 335,360 | 56.2 | 16,655,725 | 50.1 | 1,201 | 52.75 | 5,359 | 50.0 |
|  |  | Degree level or above | 175,925 | 29.5 | 9,937,885 | 29.9 | 919 | 34.83 | 4,757 | 39.0 |
|  |  | No academic or professional qualifications | 85,042 | 14.3 | 6,669,240 | 20.1 | 269 | 12.42 | 1,164 | 11.1 |
| Region | | East | 83,466 | 11.5 | 4,176,946 | 11.2 | 320 | 11.46 | 1,521 | 11.7 |
|  |  | East Midlands | 58,161 | 8.0 | 3,257,097 | 8.8 | 260 | 8.16 | 1,285 | 9.0 |
|  |  | London | 103,367 | 14.2 | 5,344,714 | 14.4 | 352 | 14.13 | 1,583 | 15.2 |
|  |  | North East | 43,454 | 6.0 | 1,847,638 | 5 | 151 | 5.34 | 650 | 5.2 |
|  |  | North West | 107,976 | 14.8 | 4,947,837 | 13.3 | 385 | 13.39 | 1,649 | 12.6 |
|  |  | South East | 110,274 | 15.2 | 6,152,088 | 16.6 | 426 | 15.06 | 2,082 | 15.8 |
|  |  | South West | 75,173 | 10.3 | 3,805,687 | 10.2 | 295 | 10.00 | 1,437 | 10.3 |
|  |  | West Midlands | 69,323 | 9.5 | 3,935,447 | 10.6 | 323 | 11.02 | 1,275 | 9.9 |
|  |  | Yorkshire and the Humber | 76,608 | 10.5 | 3,704,649 | 10 | 312 | 11.45 | 1,308 | 10.5 |
| Religious status | | Having a religion | 453,846 | 62.4 | 25,686,872 | 69.1 | 1,568 | 48.17 | 7,029 | 50.5 |
|  |  | No religion | 226,586 | 31.1 | 9,103,799 | 24.5 | 1,256 | 51.83 | 5,761 | 49.5 |
| Sex | | Female | 482,177 | 66.3 | 19,320,547 | 52 | 1,838 | 60.47 | 7,030 | 49.6 |
|  |  | Male | 245,625 | 33.7 | 17,851,556 | 48 | 986 | 39.53 | 5,760 | 50.5 |

NS-SEC = National Statistics Socio-Economic Classification, IMD = Index of Multiple Deprivation

*using a 25+ population
